# A2/A2B Deceased Donor Kidney Transplantation Using A2 Titers Improves Access to Kidney Transplantation: A Single-Center Study

**DOI:** 10.1016/j.xkme.2024.100843

**Published:** 2024-05-21

**Authors:** Erik L. Lum, Afshin Pirzadeh, Nakul Datta, Gerald S. Lipshutz, Andrea M. McGonigle, Anum Hamiduzzaman, Natalie Bjelajac, Bethany Hale-Durbin, Suphamai Bunnapradist

**Affiliations:** 1 Division of Nephrology, Department of Medicine, David Geffen School of Medicine at UCLA, Los Angeles, CA; 2 Kidney and Pancreas Transplant Research Center, Division of Nephrology, Department of Medicine, David Geffen School of Medicine at UCLA, Los Angeles, CA; 3 Departments of Surgery and Molecular & Medical Pharmacology, David Geffen School of Medicine at UCLA, Los Angeles, CA; 4 Department of Pathology, David Geffen School of Medicine at UCLA, Los Angeles, CA; 5 Department of Transplant Services, Kidney and Pancreas Transplant at UCLA, Los Angeles, CA

**Keywords:** ABO incompatible, deceased donor, kidney transplant, TK

## Abstract

**Rationale & Objective:**

The option for A2/A2B deceased donor kidney transplantation was integrated into the kidney allocation system in 2014 to improve access for B blood group waitlist candidates. Despite excellent reported outcomes, center uptake has remained low across the United States. Here, we examined the effect of implementing an A2/A2B protocol using a cutoff titer of ≤1:8 for IgG and ≤1:16 for IgM on blood group B kidney transplant recipients at a single center.

**Study Design:**

Retrospective observational study.

**Setting & Participants:**

Blood group B recipients of deceased donor kidney transplants at a single center from January 1, 2019, to December 2022.

**Exposure:**

Recipients of deceased donor kidney transplants were analyzed based on donor blood type with comparisons of A2/A2B versus blood group compatible.

**Outcomes:**

One-year patient survival, death-censored allograft function, primary nonfunction, delayed graft function, allograft function as measured using serum creatinine levels and estimated glomerular filtration rate at 1 year, biopsy-proven rejection, and need for plasmapheresis.

**Analytical Approach:**

Comparison between the A2/A2B and compatible groups were performed using the Fisher test or the χ^2^ test for categorical variables and the nonparametric Wilcoxon rank-sum test for continuous variables.

**Results:**

A total of 104 blood type B patients received a deceased donor kidney transplant at our center during the study period, 49 (47.1%) of whom received an A2/A2B transplant. Waiting time was lower in A2/A2B recipients compared with blood group compatible recipients (57.9 months vs 74.7 months, *P* = 0.01). A2/A2B recipients were more likely to receive a donor after cardiac death (24.5% vs 1.8%, *P* < 0.05) and experience delayed graft function (65.3% vs 41.8%). There were no observed differences in the average serum creatinine level or estimated glomerular filtration rate at 1 month, 3 months, and 1 year post kidney transplantation, acute rejection, or primary nonfunction.

**Limitations:**

Single-center study. Small cohort size limiting outcome analysis.

**Conclusions:**

Implementation of an A2/A2B protocol increased transplant volumes of blood group B waitlisted patients by 83.6% and decreased the waiting time for transplantation by 22.5% with similar transplant outcomes.

## Plain-Language Summary

Recipient blood type is one of the main determinants of waiting time to receive a deceased donor kidney transplant. Patients with blood type B have some of the longest waiting times for a kidney in the United States. Minorities comprise a large percentage of blood group B waitlist patients, contributing to observed racial differences in kidney transplantation rates. In this study, accepting an A2/A2B incompatible kidney resulted in receiving a kidney transplant almost 18 months earlier compared with receiving a blood group compatible kidney. No differences in outcomes were seen by accepting A2/A2B kidneys.

In 2014, the United Network for Organ Sharing (UNOS) introduced a national policy for allocation of kidneys from blood type A2 or A2B donors to blood type B recipients. This policy was introduced to improve access to deceased donor kidney transplantation for blood group B candidates, whom historically have lower transplantation rates, longer waiting times, and are more likely to be ethnic minorities.^1^, ^2^, ^3^

The A blood type contains ∼20 subgroups, of which A1 and A2 are the most common. It is estimated that 20% of blood type A patients are non-A1, of which the majority represent A2 blood type.^4^ A2 blood type antigen expression is low in kidney tissue, making it an ideal potential antigen to cross for ABO incompatible (ABOi) transplantation. Initial studies using A2/A2B kidneys demonstrated comparable allograft survival and improved access to transplantation for blood type B candidates without the need for pretransplant desensitization.^5^, ^6^, ^7^, ^8^, ^9^

Despite these reported excellent outcomes, national uptake of blood group incompatible kidney transplantation has remained low.^10^, ^11^, ^12^ Several reasons have been postulated for this low uptake, including increased risk for early rejection and allograft loss, center financial concerns because of required isohemagglutinin titer monitoring and costs of treatments such as plasmapheresis, and lack of familiarity/comfort with ABOi transplantation. An additional area of uncertainty surrounding this policy is the type of testing used to determine the risk for ABOi.^13^, ^14^, ^15^ The current policy permits centers to choose the method of measurement. Many centers use non-A1 titers as a surrogate for A2 reactivity, which may not accurately represent risk.^16^^,^^17^ Using A2 titers may be more specific and allow for improved risk assessment. However, current A2 assays are not standardized, and this may result in differences in reported outcomes between transplant centers.^18^

In this single-center study, we aimed to determine the safety and effect of A2/A2B transplantation on deceased donor kidney transplantation using an A2 titer threshold of ≤1:8 IgG and ≤1:16 IgM in blood type B recipients.

## Methods

### Study Population

This retrospective single-center cohort analyses included all deceased donor kidney transplant recipients with blood type B from January 1, 2019, corresponding to the start of the center’s A2/A2B program, to December 31, 2022. A total of 104 recipients were included, with 49 receiving an A2/A2B kidney allograft and 55 receiving a blood group compatible deceased donor kidney (B to B). Data from the Organ Procurement Transplantation Network/United Network for Organ Sharing (OPTN/UNOS) database as of March 2023 were used to provide a comparison of our center data to national data. For national data, there were total of 1,325 deceased donor kidney transplants (DDKTs) designated as having undergone A2/A2B to B deceased donor transplantation between January 2015 and December 2020. December 2020 was used as a cutoff for national analysis to provide adequate follow-up time for outcome analysis.

Clinical data were collected through retrospective chart review for single-center transplant recipients and reported data from OPTN/UNOS, which included recipient demographics (age, sex, race, end-stage renal disease cause, dialysis time, calculated panel reactive antigen, previous transplant, diabetes mellitus, hypertension, pre-emptive transplant, human leukocyte antigen mismatches, and induction immunosuppressant), donor characteristics (kidney donor profile index [KDPI], cold ischemia time, donation after cardiac death [DCD], terminal creatinine level, delayed graft function [DGF], and hepatitis C virus status) and post-transplant clinical outcomes.

Written informed consent for A2/A2B to B DDKT was obtained at time of initial UNOS listing for A2/A2B to B DDKT and again at the time of transplant. This study protocol was approved by the University California-Los Angeles institutional review board (#IRB 12-000991).

### Titer Methods

A2 IgG and IgM titers were performed using A2 reagent red blood cells (Affirmagen, Ortho-Clinical Diagnostics, Raritan, NJ) with the manual gel card method. IgG A2 titers were performed using an ant-IgG gel card (Ortho-Clinical Diagnostics, Raritan, NJ). IgM A2 titers were performed using a buffered gel card (Ortho-Clinical Diagnostics, Raritan, NJ). Serial dilutions were made using saline as the diluent. The buffered gel cards were incubated for 15 minutes at room temperature. The IgG gel cards were incubated for 15 minutes at 37°C.

### Single-Center A2B Protocol

Eligibility criteria for A2 to B transplant at our center include anti-A2 IgG antibody titer result ≤1:8 and anti-A2 IgM antibody titer result ≤1:16. All blood type B candidates underwent screening. Qualified candidates were brought back for confirmatory testing, counseling, and consent. Following consent and confirmation of acceptable titers, patients were entered into UNET for acceptance of A2/A2B deceased donor kidneys. Individuals who did not qualify or consent were maintained on the B blood group deceased donor list.

Anti-A2 titers were measured every 6 months on all actively wait listed potential A2/A2B candidates. If at any point the anti-A2 antibody titer was out of the acceptable range, the candidate was removed from A2 to B transplant list and maintained on B blood group list.

Anti-A2 antibody titers obtained at the time of admission for transplant before the operating room to confirm qualification at time of transplant. If the anti-A2 antibody titer result was out of acceptable range, the transplant was cancelled. The patient was subsequently removed from the A2 to B transplant list, but still maintained on the B blood group list.

In addition to standard post-transplant laboratory testing, recipients of A2/A2B organs underwent daily anti-A2 titers for first 9 days post-transplant. Depending on titer changes and renal function, every other day testing was then continued until post-transplant day 13.

In the case of an increasing anti-A2 titer, patients were medically managed under the discretion of the treating nephrologist. Patients could undergo plasmapheresis (PLEX) as indicated for a rapid increase in A2 titers of >4x in 24 hours, renal allograft dysfunction without clear explanation, or with biopsy-proven rejection consistent with ABOi. Biopsy was not required to initiate treatment. In patients with ongoing renal impairment despite 2-3 sessions of plasmapheresis, patients could receive eculizumab or undergo splenectomy as salvage therapy.

### Outcomes Measures

The study populations were analyzed to determine outcomes depending on the type of deceased donor kidney transplantation. The primary outcomes of interest were 1-year patient and death-censored graft survival. Secondary outcomes included delayed graft function, defined as dialysis within the first week post-transplantation; duration of DGF; primary nonfunction, defined as permanent loss of allograft function within 90 days of transplantation; serum creatinine levels at 1-year post-transplantation; biopsy-proven acute rejection, cytomegalovirus viremia; BK viremia; requirement for PLEX; and need for splenectomy post-transplantation.

### Data Analysis

Patients’ demographics were compared using descriptive statistics, including counts and percentages for categorical variables and mean and standard deviation for numerical variables. Comparison between the A2/A2B and compatible groups were performed using the Fisher test or the χ^2^ test for categorical variables and the nonparametric Wilcoxon rank-sum test for continuous variables. STATA version 16 (Statacorp, College Station, TX) was used for all statistical analyses.

## Results

### Demographics of Blood Type B Deceased Donor Kidney Transplant Recipients

A total of 104 blood type B patients received a kidney transplant at our center during the study period, 49 (47.1%) of whom received an A2/A2B transplant (Table 1). The average age of recipients who underwent A2/A2B transplantation was significantly younger compared with those receiving blood type B deceased donor kidneys (51.7 vs 58.5 years, *P* = 0.001). Men comprised a majority of transplant recipients in both groups, (67.4% in A2/A2B and 58.1% in B). A significant majority of transplant recipients were minorities, with only 8% and 7%, respectively, in the A2/A2B and B groups identified as White. No differences in cause of end-stage renal disease, pre-emptive transplantation, prior kidney transplant, or calculated panel reactive antigen were observed the between groups. A significant decrease in dialysis time was observed, with a median dialysis waiting time of 57.9 months in the A2/A2B patients compared with 74.7 months in blood group B compatible patients (*P* = 0.01).

**Table 1:** Demographics of Deceased Donor and Kidney Transplant Recipients

Characteristic	A2B (n = 49)	B (n = 55)	*P* Value
Age, y			
Mean (SD)	51.7 (11.4)	58.5 (11.5)	0.001
Range	29-70	27-77	
Sex, female, n (%)	16 (32.6%)	23 (41.8%)	0.4
Race n (%)			
Latino	19 (38.8%)	21 (38.2%)	
Asian	19 (38.8%)	21 (38.2%)	1.0
Black	7 (14.3%)	9 (16.3%)	
White	4 (8.1%)	4 (7.3%)	
ESRD cause n (%)			
DM	17 (34.7%)	17 (30.9%)	
HTN	9 (18.3%)	11 (20%)	
Unknown	2 (4.1%)	10 (18.2%)	0.1
Other	21 (42.9%)	17 (30.9%)	
Pre-emptive, n (%)	1 (2.0%)	2 (3.6%)	0.6
Previous transplant, n (%)	1 (2.0%)	4 (7.2%)	0.2
cPRA, %, mean (SD)	14.4 (26.4)	27.8 (36.3)	0.2
Dialysis time			
Mean (SD), minutes	57.9 (23.6)	74.7 (41.8)	0.01
Range, minutes	0-96	0-198	
Cold ischemia time, mean (SD)	20.9 (6.5)	22.1 (8.1)	0.7
Donor DM, n (%)	20 (41.6%)	25 (45.5%)	0.7
Donor HTN, n (%)	46 (93.9%)	53 (96.4%)	0.9
DCD, n (%)	12 (24.5%)	1 (1.8%)	<0.0001
Mismatches, n, median (IQR)	6 (5-6.5)	6 (4-7)	0.8
Terminal Cr, mg/dL, mean (SD)	1.26 (0.87)	1.81 (1.59)	0.04
KDPI, %, mean (SD)	46 (26)	56 (26)	0.05
KDPI > 85, n (%)	2 (4.1%)	13 (23.6%)	0.005
ATG, n (%)	48 (100%)	45 (81.8%)	0.002
Basiliximab, n (%)	0	10 (18.2%)	0.001

Abbreviations: cPRA, calculated panel reactive antigen; DM, diabetes mellitus; HTN, hypertension; IQR, interquartile range; SD, standard deviation.

### Deceased Donor Demographics

The mean KDPI did not differ significantly between the 2 groups, but there was a significantly higher utilization of KDPI > 85% kidneys in blood type B compatible recipients (23.6% vs 4.1%, *P* = 0.005). No differences were observed in cold ischemia time, donor diabetes, donor hypertension, and number of donor-recipient mismatches. The donor terminal creatinine level was lower in the A2/A2B recipients, and donors were more likely to be DCD (24.5% vs 1.8%, *P* < 0.05). All A2/A2B recipients received antithymocyte globulin (ATG) as induction immunosuppression per protocol, compared with 80% of blood group compatible recipients who received ATG.

### Outcomes of Blood Type B Deceased Donor Kidney Transplant Recipients

**Figure 1:**
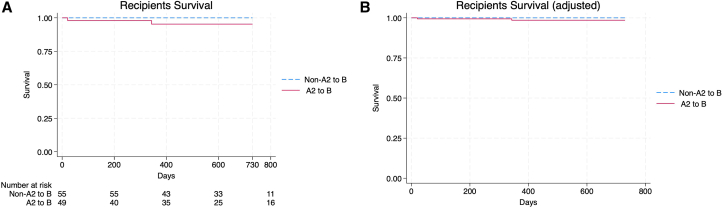
Patient survival. (A) Unadjusted analysis. (B) Adjusted analysis.

Median follow-up time was 604 days in A2/A2B recipients and 646 days in blood type B recipients (*P* = 0.96). Outcomes of transplant recipients are shown in Table 2. A total of 3 patients died in the A2/A2B group during the follow-up period (at days 21, 343, and 1,007, separately) compared with none in the blood group B compatible group; this was not statistically significant (*P* = 0.06). All patient deaths were infection related. One patient died at 21 days from a donor derived infection resulting in anastomosis break down and hemorrhage. Another patient died 11 months post-transplant from pulmonary mucor. The third patient died of disseminated cryptococcus 2 years and 9 months post-transplant. There was no observed difference in patient survival in unadjusted and adjusted models (Fig 1).

**Table 2:** Outcomes of Kidney Transplant Recipients

	A2 to B (n = 49)	Non-A2 to B (n = 55)	*P* Value
Outcomes			
Follow-up time (days) Median (IQR)	604 (356-1046)	646 (723-1622)	
Survival, n (%)	46 (93.8%)	55 (100%)	0.06
Cr month 1, mg/dl Mean (SD)	2.09 (1.44)	1.73 (1.01)	0.07
Cr month 3, mg/dl Mean (SD)	1.55 (0.72)	1.34 (0.38)	0.3
Cr year 1, mg/dL Mean (SD)	1.69 (0.68)	1.43 (0.44)	0.2
GFR month 1, mL/min	45 (35-62)	48 (37-66)	0.9
GFR month 3, mL/min	56 (45-69)	57 (42-74)	0.06
GFR year 1, mL/min	58 (43-69)	49 (41-71)	0.2
Acute rejection, n (%)	3 (6.1%)	9 (16.3%)	0.1
PNF, n (%)	1 (2%)	0 (0%)	0.3
DGF, n (%)	32 (65.3%)	23 (41.8%)	0.01
DGF days Mean (SD)	10.2 (10.2)	6 (6.5)	0.05
CMV-treated, n (%)	19 (38.7%)	23 (41.8%)	0.8
BKV-treated, n (%)	11 (22.4%)	19 (34.5%)	0.2
PLEX, n (%)	15 (30.6%)	3 (5.4%)	0.001
Transplant nephrectomy, n (%)	1 (2.0%)	2 (3.6%)	0.2
Splenectomy, n (%)	2 (4.1%)	0 (0%)	0.1

Abbreviations: BKV, BK virus; CMV, cytomegalovirus; GFR, glomerular filtration rate; IQR, interquartile range; PNF, primary nonfunction; SD, standard deviation.

**Figure 2:**
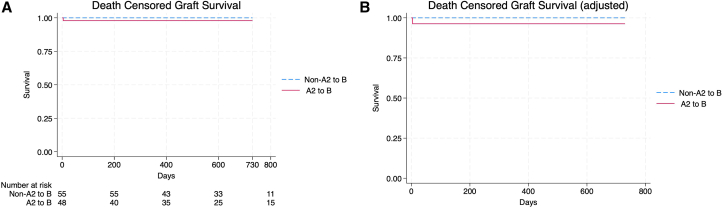
Death-censored graft survival. (A) Unadjusted analysis. (B) Adjusted analysis.

Transplant renal function with similar between the 2 groups, with no observed differences in the average serum creatinine at 1 month, 3 months, and 1 year post kidney transplantation, acute rejection, or primary nonfunction. The mean serum creatinine levels at 1, 3 and 12 months were 2.09, 1.55, and 1.69 mg/dL in the A2/A2B group compared with 1.73, 1.34, and 1.43 mg/dL in the blood type B group, respectively (*P* = 0.07, 0.25, and 0.23, respectively). The mean estimated glomerular filtration rates at 1, 3, and 12 months were 45 mL/min, 56 mL/min and 58 mL/min in the A2/A2B group compared with 48 mL/min, 57 mL/min, and 49 m:/min in the blood type B group, respectively (*P* = 0.92, 0.06, and 0.20, respectively). DCGF was similar in both unadjusted and adjusted analyses (Fig 2). DGF was more commonly observed in the A2/A2B group (65.3% vs 41.8%, *P* = 0.01). The rate of primary nonfunction was 2%, which was not significant. No statistically significant difference in biopsy-proven rejection was seen between the 2 groups; however, PLEX was more commonly used in the A2/A2B group (30.6% vs 5.4% *P* = 0.01). Two patients in the A2/A2B group required splenectomy for refractory rejection (*P* = 0.13). One patient in the A2/A2B group developed hyperacute rejection requiring transplant nephrectomy; he subsequently underwent a blood group compatible kidney transplant 12 months later and continues to have excellent allograft function. Rates of BK and cytomegalovirus viremia were similar between the 2 groups.

### Outcomes of A2B DDKTs in United States

A total of 1,325 A2/A2B to B kidney recipients were identified in the UNOS database. Patient and donor are summarized in Table S1. The median age of donors and recipients were 41.7 and 56.0 years, respectively. In addition, 34% of the recipients were female, and 43% were African American. The median dialysis time was 43.6 months, and 4.4% had a previous kidney transplant. The rate of DGF was 28.8% overall, 27.9% in patients who received a kidney with KDPI < 85%, and 40% in those who received a kidney with KDPI > 85%. The rate of primary nonfunction was 1.2% overall, 1% in patients who received a kidney with KDPI < 85%, and 4.2% in those who received a kidney with KDPI > 85%. Median serum creatinine levels after 1 year were 1.3 mg/dL overall, 1.3 mg/dL in patients who received kidney with KDPI < 85%, and 1.7 mg/dL in patients who received kidney with KDPI > 85%.

## Discussion

This study confirms the relative safety of A2/A2B to B deceased donor transplantation and demonstrates significant potential benefits of a protocol for blood group B candidates awaiting deceased donor kidney transplantation. Implementation of an A2/A2B to B deceased donor kidney transplant protocol at this single center resulted in an 83.6% increase in transplantation for blood group B candidates during the study period with comparable patient survival, death-censored graft survival, and kidney transplant function to blood group compatible transplant recipients. The increase in transplant volumes was accompanied by a marked reduction in waiting time among A2/A2B recipients (mean values of 74.7 months for B recipients and 57.9 months for A2B recipients). Longer duration of dialysis is associated with higher mortality, reduced rate of transplantation, and reduced graft survival. The reduction in waiting time by 22.5% noted in our study could potentially reduce post-transplant mortality and improve long-term graft survival.^19^ These results mirror published reports from other centers.^20^^,^^21^

The significant increase in transplantation of B group waitlisted candidates in this study directly addresses one of the key components recently laid out by the Department of Health and Human Services to increase kidney transplantation in the United States. Over 90% of the patients transplanted in our study were minorities, a group with historically low transplant rates. Implementation of an A2/A2B protocol at our center markedly improved access to kidney transplantation among this group, with an increase in transplantation rate and reduction in waiting time. Increasing national uptake of A2/A2B protocols among kidney transplant centers would further improve access and transplantation of minority candidates, who rely on deceased donor transplantation to a greater degree than their White counterparts. Our results are concerning given the low uptake of A2/A2B transplantation within the United States given that lack of availability may significantly disadvantage waitlist candidates depending on their site of listing. Furthermore, recent studies on patient preferences highlight the importance of waiting time as a major concern for waitlisted candidates and their selection of a kidney transplant center.^22^

One of the major concerns with adopting an A2/A2B protocol is an increase in transplant costs. Candidates require additional non-A1 titer screening pretransplant as well as additional post-transplant screening with our protocol. Post-transplant complications result in additional significant costs to the transplant program. Higher rates of DGF, ATG utilization, PLEX, and splenectomy are incurred by the transplant program. These financial and patient risks may deter transplant programs from adopting an A2/A2B protocol. The cost burden may be further exacerbated by a large influx of such transplants, which was shown in our study. However, increased volumes of ABOi transplants may provide increased experience to transplants centers unfamiliar with such transplants. Larger studies using more cost-efficient strategies or policies to increase monetary reimbursement for these transplants may aid in improving A2/A2B uptake.

Although overall patient survival, death-censored graft failure, and transplant allograft function were similar for A2/A2B and blood group compatible kidney recipients some noticeable differences were present, especially within the first 2 weeks of transplantation. Recipients in this single-center study experienced a significantly greater degree and duration of DGF (65.3% with an average duration of 10.2 days vs 41.8% and 6 days, respectively, for recipient of blood group compatible kidneys), although this did not affect renal allograft function at 1 month, 3 months and 1 year after transplantation. This may be partially explained by the higher rate of observed DCD donors in the A2/A2B cohort (25% vs 1.8%), a known risk factor for DGF. No differences were observed between the 2 cohorts in terms of cold ischemia time, and the A2B cohort had a lower terminal creatinine, which are factors associated with a lower risk for DGF. Although overall biopsy-proven rejection rates were lower in the A2/A2B group (6.2% vs 16%), perhaps as a result of higher ATG utilization, a significant proportion of A2/A2B recipients received plasmapheresis for increasing A2 titers (30.6%), and 3 patients experienced significant rejection episodes within the first 2 weeks of transplantation. Of the 3 recipients who experienced rejection, one resulted in early allograft loss, requiring return to dialysis and relisting, and 2 required splenectomy because of rejection refractory to plasmapheresis. The 2 patients who underwent splenectomy went on to recover allograft function. These complications highlight the need for close monitoring during the first 2 weeks following A2/A2B transplantation and experience with ABOi incompatible kidney transplantation, which may prohibit successful implementation of an A2/A2B protocol at a transplant center not well versed in ABOi transplantation or without the necessary infrastructure for close post-transplant monitoring and treatment.

Another key finding was the lower use of KDPI > 85% kidneys in A2/A2B recipients, which may have influenced serum creatinine levels and graft survival. However, adjusted analysis showed no differences between groups. Despite these risks, the marked improvements in transplant access and excellent long-term outcomes seen in this study suggest major advantages for those candidates who qualified and received a kidney transplant under the A2/A2B policy at our center.

The available pool of DDKTs has increased over the past several years. However, the deceased donor organs remain a finite and limited resource, and transplantation for one group does not exist in a vacuum independently of another group. The significant increase in kidney transplantation seen in our study presumably comes at the cost of diverting A2 and A2B kidneys from A and AB waitlisted candidates who do not have an immunological barrier to transplantation of these organs. This would presumably increase the wait times for a transplant for these candidates. However, 1-year death-censored graft function was not different in our study cohort. These results indicate that although there is an immunological barrier to A2/A2B transplantation kidney, outcomes were not affected. We found a trend, although not significant, toward lower KDPI for those receiving an A2/A2B incompatible transplant, indicating that organs of higher quality are possibly being transplanted into A2/A2B compared with blood group compatible recipients (46% vs 56%, respectively). Although this benefited the A2/A2B recipients, the removal of higher quality organs from the A group waitlist may be a cause for concern. One potential alternative would be to divert some blood group O deceased donor kidneys to B blood group candidates, thus obviating the increased risk of ABOi incompatibility. However, this may significantly adversely affect blood group O candidates, who can only receive kidneys from O blood group donors.

This single-center study has several limitations. The major limitation of our study is its small sample size combined with the small number of observed complications, which limits the analysis of post-transplant complications and assessment of post-transplant IgM and IgG titer changes. Another limitation is our use of A2 titers. Significant variability in A2 titers levels has been reported between institutions, with some studies demonstrating upward of a 4-fold difference. This difference could result in significant differences in outcomes between transplant centers and partially explain the excellent outcomes seen in our study compared with other centers using the same A2 cutoffs. It is notable that there were 3 episodes of severe ABOi-related rejection, indicating that the protocol was not without risk. However, comparing our outcomes to national outcomes in A2/A2B transplantation, in which many centers use A1 titers as a surrogate, yields similar results (Table S1). Another limitation in our study was the high use of plasmapheresis. Over one-third of patients experienced an increase in A2 titers and received pre-emptive plasmapheresis. Not all patients received a kidney biopsy. In addition, given the high incidence of DGF in the study, the decision for plasmapheresis was determined by the physician caring for the patient during transplantation. It is unclear whether pre-emptive plasmapheresis was beneficial or what level of titer increase is cause for concern from this study.

Future studies involving larger cohorts and standardized or centralized A2 testing would be beneficial in confirming the safety of using A2 titers for qualified blood group B candidates awaiting deceased donor kidney transplantation and provide crucial information on titer trends after transplantation. Studies using higher titer cutoffs are needed to determine whether more B blood group candidates could be listed for A2 kidneys without an increase in complications.

In conclusion, implementation of A2 to B and A2B to B with a protocol using A2 titers of ≤1:8 for IgG and ≤1:16 for IgM in a single center significantly increased deceased donor kidney transplant rates and reduced waiting times in blood group B waitlist candidates without reducing 1-year patient survival, death-censored graft survival, or graft function. Implementation of such protocols should be strongly considered by transplant programs to improve access to transplantation for their waitlist B blood type candidates.
