# Supplementary material for: A2/A2B Deceased Donor Kidney Transplantation Using A2 Titers Improves Access to Kidney Transplantation: A Single-Center Study
Source: Kidney Med. 2024 May 21;6(7):100843. doi: 10.1016/j.xkme.2024.100843 (PMC11214338; doi:10.1016/j.xkme.2024.100843)
Supplement: Supplementary File (PDF) — Table S1. [file mmc1.docx]

|  |  |
| --- | --- |
| Donor characteristics |  |
| Median age, years (IQR) | 43 (31-53) |
| Female, n (%) | 553 (41.7%) |
| African American, n (%) | 137 (10.3%) |
| Smoking, n (%) | 334 (25.2%) |
| Hypertension, n (%) | 452 (34.1%) |
| Diabetes, n (%) | 101 (7.6%) |
| CMV positive, n (%) | 732 (55.2%) |
| HCV positive, n (%) | 91 (6.8%) |
| Median terminal serum creatinine, mg/dl (IQR) | 1 (0.7-1.4) |
| Cerebrovascular cause of death, n (%) | 404 (30.4%) |
| Donation after cardiac death, n (%) | 341 (25.7%) |
| Median KDPI, % (IQR) | 46 (25-66) |
| Donor with KDPI ≥85%,  n (%) | 95 (7.1%) |
| Recipients characteristics |  |
| Median age, years (IQR) | 56 (47-65) |
| Female, n (%) | 455 (34.3%) |
| African American, n (%) | 576 (43.4%) |
| Diabetes, n (%) | 545 (41.1%) |
| Peripheral vascular disease, n (%) | 168 (12.6%) |
| CMV positive, n (%) | 939 (70.8%) |
| HCV positive, n (%) | 55 (4.1%) |
| Cause of ESRD, n (%) |  |
| Glomerular diseases | 194 (14.6%) |
| Diabetes | 459 (34.6%) |
| Hypertension | 332 (25.1%) |
| Others | 340 (25.7%) |
| Preemptive kidney  transplant, n (%) | 164 (12.3%) |
| Median dialysis vintage, days (IQR) | 1,310  (717-1,917) |
| Previous kidney transplant, n (%) | 59 (4.4%) |
|  |  |
| Machine perfusion pump used, n (%) | 650 (49%) |
| Median HLA mismatch, (IQR) | 5 (4-5) |
| Median peak PRA, % (IQR) | 0 (0-3) |
| Induction immunosuppression, n (%) |  |
| None | 79 (5.9%) |
| Antithymocyte globulin | 849 (64.1%) |
| Anti-IL-2R antibody | 160 (12.1%) |
| Alemtuzumab | 170 (12.8%) |
| Combined | 18 (1.3%) |
| Others | 49 (3.6%) |
| Outcomes |  |
| Overall delayed graft function, n (%) | 382  (28.8%) |
| KDPI <85% | 344/1,230  (27.9%) |
| KDPI ≥85% | 38/95  (40%) |
| Overall primary non-function, n (%) | 17  (1.2%) |
| KDPI <85% | 13/1,230  (1%) |
| KDPI ≥85% | 4/95  (4.2%) |
| Overall median serum creatinine at 1-year post-transplant, mg/dl (IQR) | 1.3  (1.1-1.6) |
| KDPI <85% | 1.3  (1.1-1.6) |
| KDPI ≥85% | 1.7  (1.3-2.1) |
